# Minimally invasive clamp-assisted reduction and long InterTAN nail fixation for Seinsheimer type V subtrochanteric fractures: a case series describing the technique and results

**DOI:** 10.1186/s12891-023-06363-4

**Published:** 2023-04-03

**Authors:** Zhen Wu, Bin Du, Qiang Wang, Tao Jiang, Yincong Si, PanJun Zhang, Yong Wang

**Affiliations:** Department of Orthopedic Surgery, the Affiliated Yixing Hospital of Jiangsu University, No. 75 Tongzhenguan Road, Yixing, 214200 Jiangsu, P.R. China

**Keywords:** Subtrochanteric fracture, Intertrochanteric fracture, Classification, Minimally invasive, Clamp-assisted reduction, Intramedullary nail

## Abstract

**Background:**

Surgical treatment of Seinsheimer type V subtrochanteric fractures is extremely challenging due to the difficulty of obtaining and maintaining anatomic reduction and effective fixation. The purpose of this study was to describe a surgical technique for minimally invasive clamp-assisted reduction and long InterTAN nail fixation to manage Seinsheimer type V subtrochanteric fractures and report the clinical and radiological results.

**Methods:**

A retrospective study was conducted on patients with Seinsheimer type V subtrochanteric fractures between March 2015 and June 2021. A total of 30 patients treated via minimally invasive clamp-assisted reduction, long InterTAN nail fixation and selective augmentation with a cerclage cable were included. The following data were collected and evaluated: patient demographics, operative time, blood loss, reduction quality, tip apex distance (TAD), time to bone union, Harris hip score (HHS), visual analog score (VAS), and complications.

**Results:**

The mean age of the 30 patients was 64.8 years (range: 36-90 years). The mean operative time was 102.2 min (range: 70-150 min). The mean loss of blood was 318.3 ml (range: 150-600 ml). The reduction quality involved 27 cases of anatomic reduction and 3 cases of satisfactory reduction. The mean TAD was 16.3 mm (range: 8-24 mm). The mean follow-up time was 18.9 months (range: 12-48 months). The mean fracture healing time was 4.5 months (range: 3-8 months). The mean Harris score was 88.2 (range: 71-100), and the VAS score was 0.7 (range: 0-3). Delayed union of the subtrochanteric fracture site occurred in two patients. The limb length discrepancy, which was determined in 3 patients, was < 10 mm. There were no significant complications.

**Conclusion:**

Our results indicate that minimally invasive clamp-assisted reduction with long InterTAN nail fixation is encouraging for Seinsheimer Type V subtrochanteric fractures, resulting in excellent reduction and fixation. Additionally, this reduction technique is simple, reliable, and effective in reducing and maintaining subtrochanteric fractures, particularly when intertrochanteric fractures are irreducible.

## Background

Subtrochanteric fracture is defined as a major fracture line within 5 cm below the lesser trochanter, accounting for 25% of hip fractures [1]. Many classification systems that aim to characterize the severity and guide the treatment of subtrochanteric fractures currently exist, including the Seinsheimer classification, which is widely used in clinical fields. This classification categorizes subtrochanteric fractures into five types based on the number of fractured fragments, location, and shape of the fracture line [2, 3]. Of these five types, Type V involves subtrochanteric and intertrochanteric spaces and is known as a complex and unstable fracture [3]. Surgical management of Type V fractures is extremely challenging due to the difficulty of obtaining and maintaining anatomic reduction and effective fixation [3].

The displacement of the subtrochanteric fracture is obvious owing to the strong deforming forces of the surrounding muscles and ligaments; hence, it is difficult to achieve anatomic reduction—a key factor in achieving the best results in subtrochanteric fractures—via closed reduction [4]. For Type V fractures, it is more challenging to perform a reduction when intertrochanteric fractures fail by the closed method. Some have suggested using open reduction maneuvers to improve the quality of reduction; however, the morbidities associated with this technique, such as increased blood loss, operative time, and nonunion rate due to periosteal damage, remain a major concern [5]. To achieve adequate reduction and minimize disturbing the periosteal blood supply, multiple minimally invasion-assisted reduction methods and tools, such as ball spike pushers, bone hooks, pointed clamps, and cerclage wires or cables, have been described [6–8]. However, most of them make it difficult to obtain and especially maintain reduction when reaming and inserting nails, even for experienced surgeons. Clamp-assisted reduction of subtrochanteric fractures, originally described by Afsari et al. [9], has shown good results with minimal complications. Subsequently, this technology has been gradually promoted for clinical application [10, 11]. However, to the best of our knowledge, few reports have described minimally invasive clamp-assisted reduction for type V subtrochanteric fractures, especially when the reduction of intertrochanteric fractures is simultaneously challenging [9–11].

The preferred surgical fixation for subtrochanteric fractures is intramedullary nailing, as it offers superior biomechanical and biological excellence compared to extramedullary devices [12]. The most commonly used intramedullary nails include reconstruction nails, Gamma 3, proximal femoral nail anti-rotation (PFNA), and InterTAN; however, the type of nail with superior clinical and radiological results is still controversial [3, 13]. Multiregion fixation and high stress tolerance must be considered when selecting intramedullary nails for Type V fractures, as they involve subtrochanteric and intertrochanteric regions simultaneously. The InterTAN nail is specifically designed for intertrochanteric fractures and has unique anti-rotation and linear pressurization advantages [14, 15]. Theoretically, it is more suitable for Type V fractures. However, few studies have described the use of InterTAN nails for the surgical treatment of Type V fractures [3].

In our study, we adopted a minimally invasive clamp-assisted reduction, long InterTAN nail fixation, and selective augmentation with a cerclage cable to manage Seinsheimer Type V subtrochanteric fractures and achieved promising results with low complication rates. Therefore, this study aimed to describe our surgical technique and report the clinical and radiological outcomes of 30 patients treated using this method.

### Patients and methods

This retrospective study was approved by the ethics committee of our hospital. Between March 2015 and June 2021, 30 consecutive patients with operatively treated Seinsheimer type V subtrochanteric fractures using minimally invasive clamp-assisted reduction, long InterTAN nail fixation, and selective augmentation with cerclage cables were enrolled in our hospital. The exclusion criteria were as follows: 1. age < 18 years; 2. old fractures; 3. open fracture; 4. pathological fractures or atypical subtrochanteric fractures; 5. fractures that were reduced successfully with closed reduction; and 6. follow-up of < 12 months.

### Surgical technique

Preoperative radiographs, including the pelvis anteroposterior (AP) view, AP and lateral view of the injured hip, were obtained. Additionally, computed tomography (CT) images of the pelvis and femoral shaft and 3D reconstruction were used to evaluate the location of the fracture, fracture configuration, and displacement feature. Additionally, CT was used to determine the planned length and diameter of the intramedullary nail.

All surgeries were performed under general or spinal anesthesia, and the patient was positioned supine on a radiolucent operative traction table. For obese patients, abdominal fat was fixed with a bandage to make it easy to obtain the right nail entry site. Then, the opposite limb was fixed in the hemilithotomy position, and the affected limb was fixed with boot traction for longitudinal traction. First, closed reduction was performed and assessed under fluoroscopy on the AP and lateral views. If successful, a standard antegrade cephalomedullary nailing technique was performed. However, in all patients, closed reduction of the subtrochanteric fractures failed. The skin incision site was chosen according to the location of the subtrochanteric fracture, confirmed under fluoroscopy. An approximately 5 cm incision was made and centered at the fracture site in the lateral and slightly posterior aspect of the thigh due to anteversion of the femoral neck and external rotation of the proximal femur to place the head-neck screw with the same incision. The fascia lata was incised, and the fibers and vastus lateralis were bluntly dissected until the fracture was palpated. Consequently, direct visualization of the fracture and peeling of the periosteum was unnecessary.

**Fig. 1:**
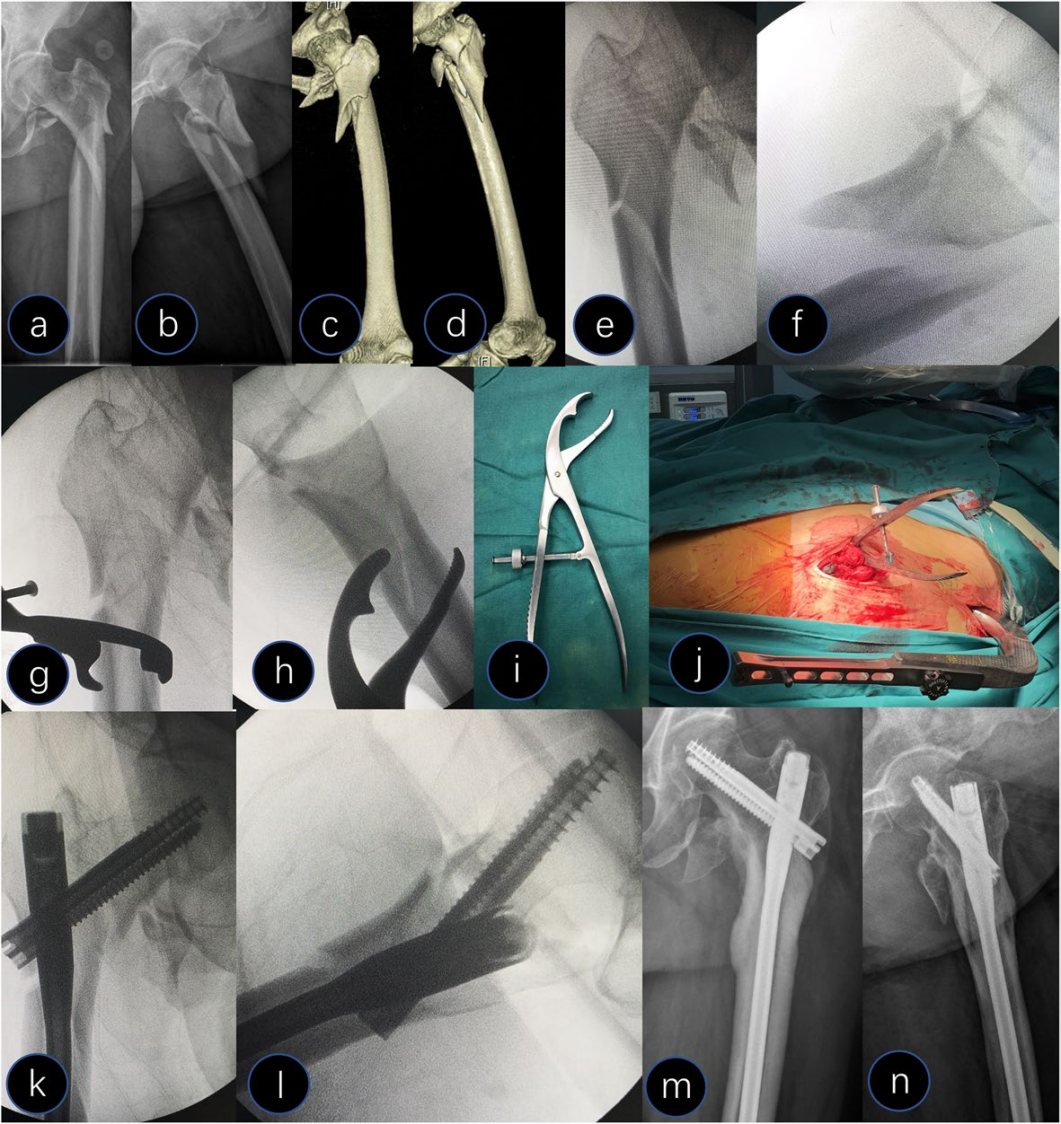
A 65-year-old female presented with a Type V fracture. **a-d** Preoperative X-ray and computed tomography 3D reconstruction showed an anterior-posterior displacement of the subtrochanteric region. **e **and** f** Closed reduction with traction under fluoroscopy. **g **and** h** A two-jaw clamp was used to reduce the subtrochanteric fracture. **i** Picture of the two-jaw clamp. **j** Surgical pictures of the clamp placement. **k **and** l** Intraoperative X-ray after reduction and fixation. **m **and** n** Postoperative X-ray at 13 months

**Fig. 2:**
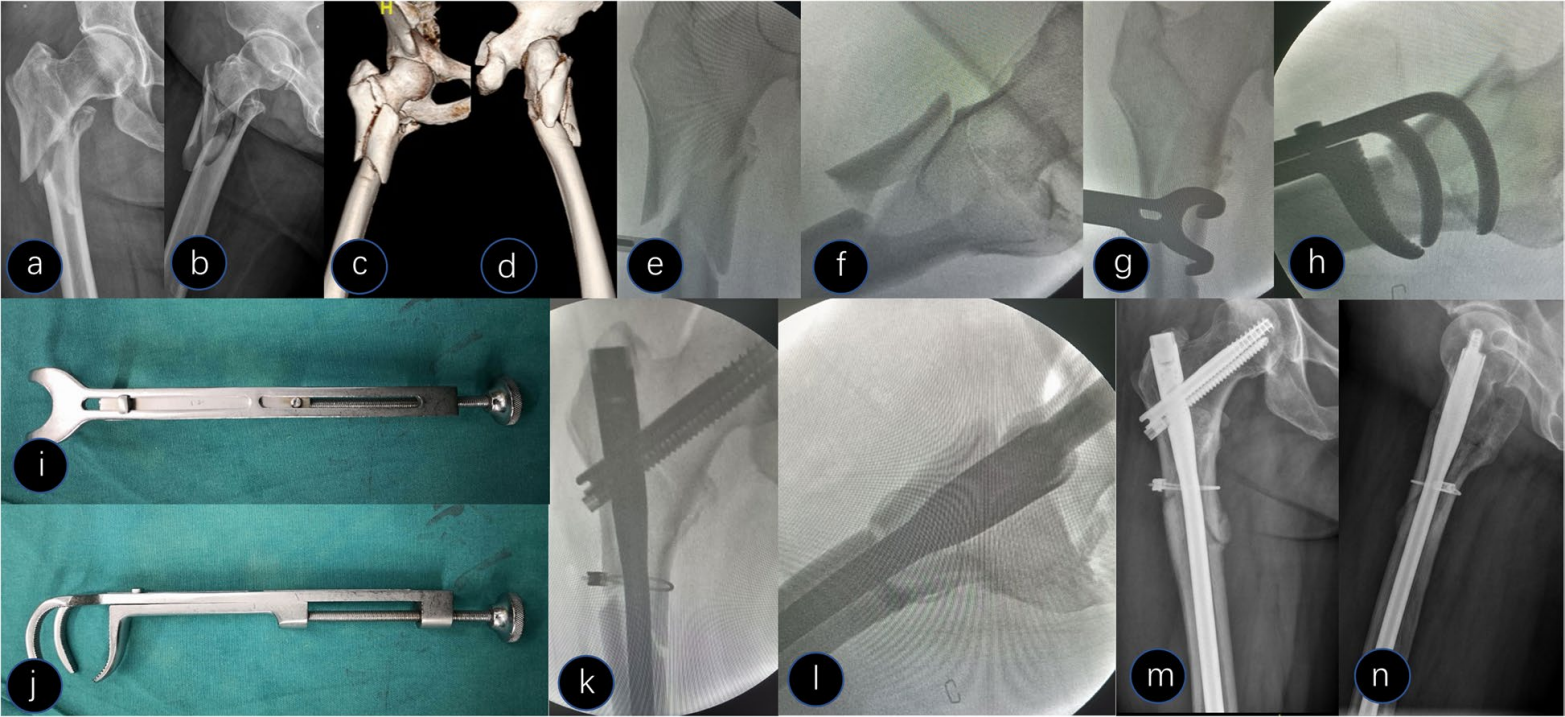
A 54-year-old female presented with a Type V fracture. **a-d** Preoperative X-ray and computed tomography 3D reconstruction showed medial-lateral displacement of the subtrochanteric region. **e **and** f** Closed reduction with traction under fluoroscopy. **g **and** h** A Lowman clamp was used to reduce the subtrochanteric fracture during the operation. **i **and** j** Pictures of the Lowman clamp. **k **and** l** Intraoperative X-ray after reduction and fixation (a cerclage cable was used to fix a large butterfly-shaped fragment). **m **and** n** Postoperative X-ray at 6 months

Next, the technique of clamp-assisted reduction was divided into two groups according to the fracture configuration and the resultant displacement of the fracture fragments. It was performed separately in the subtrochanteric region. In Group I (Fig. 1), the main fracture fragments exhibited anterior-posterior displacement in the lateral view and presented a spiral fracture pattern in which the proximal fragment was in flexion, abduction, and external rotation. First, the length and rotational alignment were restored using a traction table. Then, a two-jaw clamp was introduced through the lateral incision for reduction with the prongs and advanced until it touched the fracture site. The clamp was opened slowly with its prong grasping anterior-posterior through the fracture and compressing fragments perpendicular to the fracture plane. The prongs were opened slightly if malalignment was observed by palpation and fluoroscopy assessment. Then, the fracture site was finely manipulated to correct the length, rotation, and alignment and recompressed until anatomic reduction was achieved. At this stage, it was crucial to be particularly aware of the location of the clamp to prevent it from interfering with the combined neck-head screw insertion. Therefore, the clamp site was as far as possible from the fracture site in a satisfactory temporary fixation. In group II (Fig. 2), the main fragments exhibited medial-lateral displacement in the AP view and presented as long oblique and reverse oblique fracture patterns. Furthermore, a Lowman clamp was introduced through the lateral incision for reduction with the prongs opened and advanced from the superior surface of the bone until it touched the medial cortex of the fracture site. Next, the clamp was closed slowly with its prong grasping medial-lateral through the fracture and compressing fragments perpendicular to the fracture plane. If malalignment was observed by finger palpating and fluoroscopy assessment, the prongs were opened slightly, and the fracture site was finely manipulated to correct the length, rotation, alignment, and recompression until anatomic reduction was achieved. During this step, care was taken to avoid injury to the vascular structure on the medial side by maintaining contact with the medial cortex using one prong.

Additionally, surgeons ensured that the clamp was tightly closed until fixation with the intramedullary nail was finished in all patients. The cerclage cable selectively aided fixation, associated with a large butterfly-shaped fragment involving the lateral wall or fracture displaced after clamp removal, and the procedure through the same incision as the clamp reduction.

**Fig. 3:**
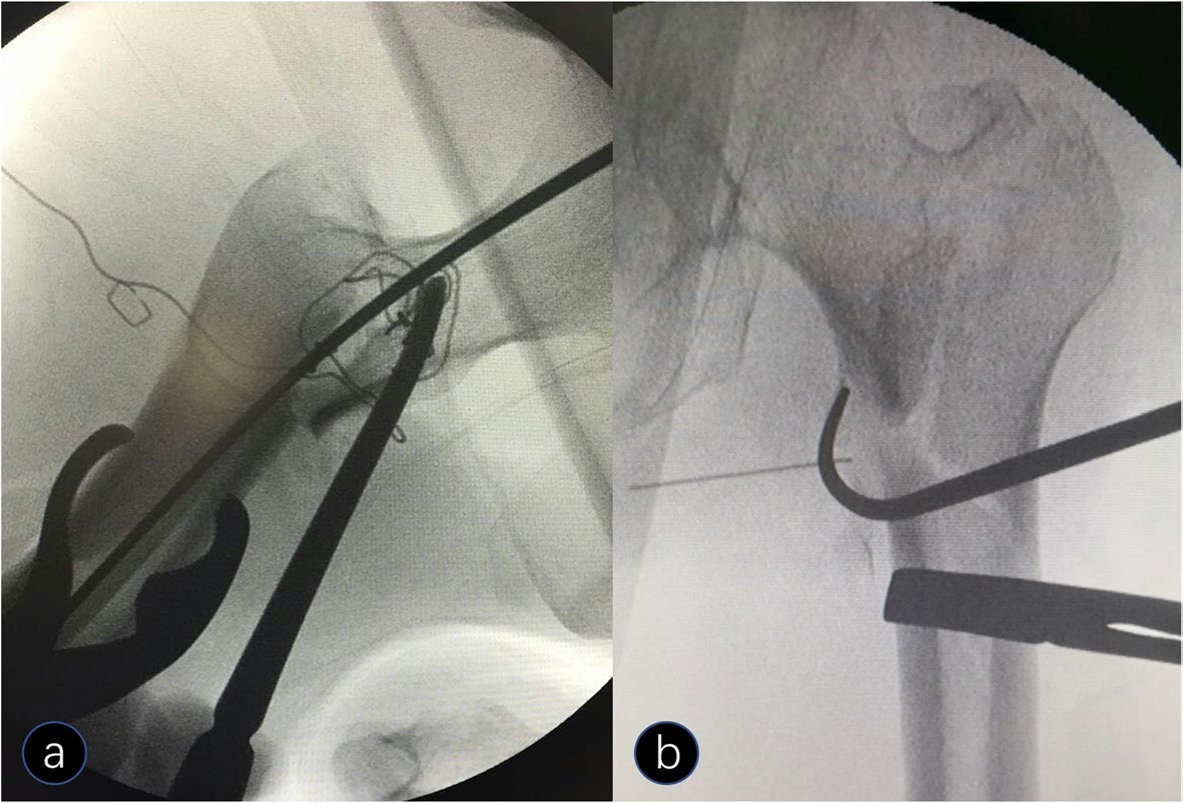
**a** A periosteal stripper applied to the anterior displacement of the proximal fragment when closed reduction of the intertrochanteric fracture failed. **b** A bone hook applied to the medial displacement of the proximal fragment when closed reduction of the intertrochanteric fracture failed

When subtrochanteric fracture reduction was completed, close reduction of the intertrochanteric fracture was achieved by longitudinal traction, adduction, and internal rotation of the affected limb. Assisted tools were used if the closed reduction failed, such as a periosteal stripper applied to the anterior displacement of the proximal fragment or a bone hook applied to the medial displacement of the proximal fragment to restore alignment with the same lateral incision for subtrochanteric fracture reduction (Fig. 3).

Once the reduction of the subtrochanteric and intertrochanteric fractures was satisfactory, an incision was made 4 cm above the tip of the greater trochanter. The entry point was slightly medial to the tip of the greater trochanter in the coronal plane and at the anterior one-third and posterior two-thirds in the sagittal plane. Then, a ball-tipped guide wire was inserted into the medullary cavity after opening the entry point. After proximal reaming, distal reaming was performed by increasing the diameter of the reamer by 0.5-1.0 mm until cortical chatter occurred. Once reaming was completed, the chosen long InterTAN nail was inserted. Additionally, a combination of sleeve and head/neck component screws were inserted, which were controlled at the center or slightly below the femoral neck on the AP and the center on the lateral view. The distal static locking screws were locked through the target device. The surgeon needed to reassess whether instability or displacement of the fracture site occurred after clamp removal by palpating or fluoroscopy. If the results were positive, a cerclage cable was placed through the subtrochanteric reduction incision for augmentation fixation. All incisions were then closed layer-by-layer.

### Postoperative management

Postoperatively, antibiotics were prophylactically administered to prevent infection, and low-dose heparin was administered to prevent deep vein thrombosis. All patients were encouraged to perform functional exercises during the early postoperative period. Partial weight-bearing with a walker was allowed after 2 weeks, and full weight-bearing was permitted only after full radiographic fracture union. Routine follow-up was recommended every 1 month until the presence of radiological consolidation and every 3 months thereafter.

Reduction quality was assessed by postoperative radiography according to the modified criteria proposed by Baumgartner [16]. TAD was related to the sum of the distances from the tip of the upper lag screw to the femoral head apex on AP and lateral views [14]. Radiographic union was defined as the presence of bone bridging in at least three of the four cortices seen in the AP and lateral radiographs during the follow-up visits [17]. Additionally, delayed union was defined as radiographic evidence of healing until six months postoperatively [4]. Nonunion was confirmed by the lack of healing after one year or by the requirement for reoperation [17]. The limb length discrepancy was measured immediately postoperatively by comparison with the uninjured limb. Then, clinical outcomes were evaluated using the Harris hip score and the visual analog scores.

## Results

The baseline data for the 30 included patients are shown in Table 1. There were 16 men and 14 women with an average age of 64.8 years (range: 36-90 years). Fracture injury mechanisms were as follows: falling from a standing height in 16 patients, traffic accidents in 6 patients, motorcycle crashes in 4 patients, and falling from a height in 4 patients. The mean BMI was 24.6 kg/m2 (range: 18.7-35.2 kg/m2). The median time to definitive surgery was 4.6 days (range: 2-8 days).

**Table 1:** Patients with Seinsheimer Type V subtrochanteric fractures

No.	Age, y	Sex	BMI, kg/m2	Method of reduction		Cerclage cable	Operative time, min	Loss of blood, ml
				Subtrochanteric	Intertrochanteric			
1	66	F	24.8	Lowman clamp	Closed reduction	No	90	300
2	36	M	20.1	Lowman clamp	periosteal stripper	Yes	110	450
3	45	M	30.7	two-jaw clamp	Closed reduction	No	100	250
4	63	M	21.7	Lowman clamp	periosteal stripper	Yes	120	400
5	76	F	33.0	two-jaw clamp	Closed reduction	No	70	300
6	53	M	26.0	two-jaw clamp	Closed reduction	No	90	200
7	69	M	29.4	Lowman clamp	Closed reduction	No	100	250
8	42	M	25.1	Lowman clamp	Closed reduction	No	110	300
9	53	M	24.8	Lowman clamp	Closed reduction	No	95	300
10	84	F	20.5	two-jaw clamp	Closed reduction	No	90	200
11	65	F	23.9	two-jaw clamp	Closed reduction	Yes	150	600
12	73	M	22.5	Lowman clamp	Closed reduction	No	85	200
13	68	M	30.1	Lowman clamp	Limited open reduction with iliopsoas muscle releasing	No	130	500
14	76	F	23.4	two-jaw clamp	bone hook	No	100	350
15	80	F	28.0	Lowman clamp	periosteal stripper	Yes	110	550
16	70	F	20.7	two-jaw clamp	Closed reduction	Yes	125	600
17	48	M	22.6	Lowman clamp	Closed reduction	Yes	105	450
18	65	M	21.5	Lowman clamp	Closed reduction	No	105	200
19	71	F	20.1	two-jaw clamp	bone hook	No	95	350
20	68	M	26.0	Lowman clamp	Closed reduction	No	100	200
21	90	F	20.8	Lowman clamp	periosteal stripper	Yes	110	450
22	55	M	28.4	Lowman clamp	Closed reduction	No	85	250
23	65	F	35.2	two-jaw clamp	Closed reduction	No	120	300
24	73	F	20.8	two-jaw clamp	Closed reduction	No	80	200
25	87	F	18.7	Lowman clamp	Closed reduction	No	70	150
26	48	M	22.9	two-jaw clamp	Closed reduction	No	110	300
27	54	F	27.5	Lowman clamp	Closed reduction	Yes	120	300
28	69	F	20.5	two-jaw clamp	periosteal stripper	Yes	70	150
29	67	M	27	two-jaw clamp	Closed reduction	No	90	200
30	65	M	22.9	Lowman clamp	Closed reduction	Yes	130	300
Quality of reduction	Time union (months)	TAD (mm)	Harris Hip Score	Visual analog score	Follow-up (months)	Complications		
AR	4	18	84	1	12	N		
AR	3	10	100	0	18	N		
AR	4	9	96	0	24	N		
SR	7	13	89	1	24	Delayed union		
AR	4	15	86	1	15	N		
AR	3	13	97	0	24	N		
AR	4	16	90	0	36	N		
SR	5	12	77	3	19	N		
SR	4	23	87	1	13	N		
AR	4	15	75	1	12	N		
AR	5	15	85	1	15	Serous drainage		
AR	4	22	87	0	16	N		
AR	6	11	92	1	48	N		
AR	4	13	93	0	18	N		
AR	5	21	81	2	18	N		
AR	8	20	79	2	28	Delayed union		
AR	3	18	100	0	22	N		
AR	5	8	95	0	30	N		
AR	4	16	86	0	15	N		
AR	4	15	90	0	18	N		
AR	6	21	71	1	15	N		
AR	3	13	95	0	12	N		
AR	4	24	96	0	18	N		
AR	5	19	92	1	15	N		
AR	4	12	78	1	12	N		
AR	3	22	90	1	15	N		
AR	6	14	87	1	15	N		
AR	5	23	91	1	14	N		
AR	4	17	92	0	12	N		
AR	5	21	86	0	15	N		

*AR* Anatomical reduction, *SR* Satisfactory reduction

**Fig. 4:**
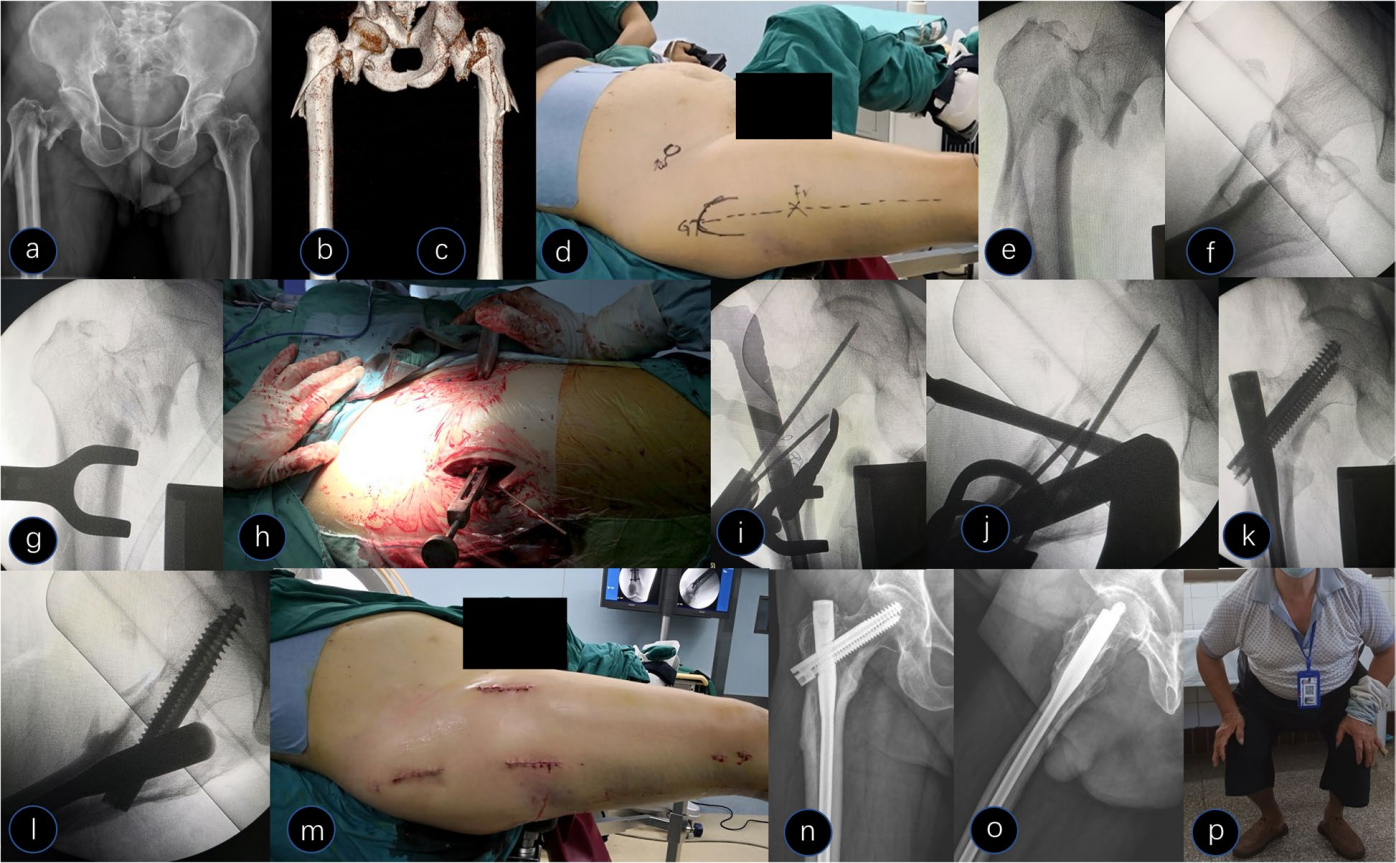
A 68-year-old male presented with a Type V fracture. **a-c** Preoperative X-ray and computed tomography 3D reconstruction showed medial-lateral displacement of the subtrochanteric region and medial-lateral displacement of the intertrochanteric region. **d** Picture of the surgical posture. **e **and** f** Closed reduction with traction under fluoroscopy. **g** A Lowman clamp was used for the subtrochanteric fracture, and the intertrochanteric fracture was irreducible (iliopsoas muscle embedded into the lesser trochanter). **h** Surgical picture of the maintained reduction in both regions. **i **and** j** Intraoperative X-ray after both regions were reduced. **k **and** l** Intraoperative X-ray after reduction and fixation. **m** Postoperative picture of the incision. **n **and** o** Postoperative X-ray at 48 months. *p* Functional picture at follow-up

Group I had 13 patients (43.3%) who presented a spiral fracture pattern, and the proximal fragment was in flexion, abduction, and external rotation. Group II had 17 patients (56.7%) who presented long oblique and reverse oblique fracture patterns. The intertrochanteric fracture could not be successfully reduced through closed reduction in eight cases. Of these, five cases associated with anterior displacement of the proximal fragment were reduced by a periosteal stripper, three cases were associated with medial displacement of the proximal fragment, and two were reduced by a bone hook. The other was a rare case in which reduction failed with a bone hook due to embedding of the iliopsoas muscle into the lesser trochanter [18] (Fig. 4). An extra anterior incision of approximately 6 cm was made to release the iliopsoas muscle and assist reduction with periosteal stripper pressure.

Among all patients, 10 had a large butterfly shaped fragment, and fixation was augmented with one cable, including the lateral and medial walls in five and four cases, respectively, and simultaneous involvement of the lateral and medial walls in one case (Fig. 2).

All surgeries were completed with no intraoperative neurovascular injury. The average operative time was 102.2 min (range: 70-150 min), with a mean blood loss of 318.3 ml (range: 150-600 ml). According to the Baumgartner criteria for reduction quality, anatomic reduction was obtained in 27 cases, and satisfactory reduction was achieved in 3 cases. The mean TAD was 16.3 mm (range: 8-24 mm). The mean length of follow-up was 18.9 months (range: 12-48 months). All fractures healed within 4.5 months (range: 3-8 months). At the last follow-up, the mean Harris score was 88.2 (range: 71-100), including 15 excellent, 10 good, and 3 fair cases; 25 of 30 (83.3%) patients achieved excellent hip function. The mean VAS score was 0.7 (range: 0-3).

Two patients had delayed union and eventually achieved healing at 7 and 8 months without further intervention. One patient had serous drainage from the surgical wound that eventually healed through dressing changes and did not require antibiotic treatment. Three patients had a limb length discrepancy of < 10 mm. There were no significant complications, such as deep infection, deep vein thrombosis, nonunion, cut-out, and implant failure.

## Discussion

Despite improving reduction methods and designing intramedullary nails, surgical treatment of Seinsheimer Type V subtrochanteric fractures remains a challenge for surgeons to obtain and maintain adequate reduction and stable fixation [3]. In this study, we adopted minimally invasive clamp-assisted reduction, long InterTAN nail fixation, and selective augmentation with cerclage cables for combined subtrochanteric and intertrochanteric fractures. Consequently, all patients achieved excellent clinical and radiological results with low complication rates.

Classic textbooks and most of the literature describe a characteristic subtrochanteric fracture deformity pattern with a short proximal fragment in flexion, abduction, and external rotation due to the forces acting on the proximal femur (iliopsoas, adductor, and gluteal muscles) and a medialized distal fragment caused by the pull of the adductors [1]. However, this pattern of deformity in subtrochanteric fractures is not constant [19]. Recently, Yoon et al. [7], Lim et al. [8] and Mingo-Robinet et al. [10] described that in addition to the above characteristic deformity pattern, there is another common deformity pattern with abduction as the main deforming force. Both patterns of deformity are difficult to reduce using the closed method. The injury cohort evaluated in our study can be similarly described. For Type V fractures, the surgeries are challenging when both fractures are irreducible types.

In the present study, we separately selected a two-jaw or Lowman clamp for reduction according to the fracture pattern and displacement of the fracture fragments. All patients achieved satisfactory reduction and a high anatomical reduction rate of 90%. From our results, the advantages of clamp-assisted reduction are as follows. First, the clamp is an inexpensive and widely available tool in various hospitals. Second, it is relatively easy to use and facilitates the improvement of the quality of reduction. In particular, the Lowman clamp was beneficial for encompassing fracture fragments (Fig. 2). Third, it can make the correct entry point more accurate, particularly for obese patients whose legs can be adducted, to facilitate the approach to the correct nail entry point. Fourth, assistants are not required to maintain reduction during canal preparation and nailing. Fifth, the clamp is not loose during reaming and nail insertion, which is critically important because most minimally invasive reduction tools remain problematic. Sixth, this is particularly useful when both the subtrochanteric and intertrochanteric fracture patterns are irreducible, and we believe this procedure has not been described in the literature. Surgery is complex when this combination occurs. This problem is solved effectively with our method, which causes the two-part fracture to shift to a one-part fracture with a clamp, and the subtrochanteric fracture is undisturbed with clamp fixation during the entire procedure. In our group, there were 8 cases of irreducible intertrochanteric fractures; we maintained the subtrochanteric region with a tight clamp and reduced the intertrochanteric fracture with assisted tools (Fig. 4).

Despite the above advantages of clamp-assisted reduction, we acknowledge that the technique has some limitations, including the disruption of the closed fracture environment, which may theoretically lead to higher rates of infection and decreased union if local biology is compromised. We attempted to reduce the damage to the periosteum as much as possible. Mingo-Robinet et al. [10] reported head-neck screw insertion interference with clamping as the most common problem. We encountered a similar case, and although the reduction was not lost during readjustment, we suggested that the clamp be positioned as far as possible from the proximal fracture site. Inappropriate use of the clamp, especially the Lowman clamp, can be detrimental to neurovascular structures.

Eventually, reliable fixation is mandatory when all the reduction steps are completed. Because Type V fractures involve a larger span and have extremely high mechanical stresses, complications, such as varus collapse, implant cut-out, and implant breakage, remain a risk. [3] Hence, the correct choice of the intramedullary nail must ensure sufficient stability to achieve satisfactory outcomes and prevent complications. Park et al. [17] noted that a large cephalic screw inserted toward the femoral head provides secure angular and rotational stability in subtrochanteric fractures. The InterTAN nail provides excellent angular and torsional stability at the proximal segment because of its two interlocking screws inserted into the femoral head. Moreover, the proximal end of the InterTAN nail has a trapezoidal shape, which results in more material being present on the lateral side of the implant, improving the ability of the lateral wall to support and resist stress [14]. Several biomechanical studies indicate that InterTAN nails have more advantages in terms of strength, stability, and resistance to bending and rotation than Gamma 3 and PFNA nails [15]. Additionally, clinical research has confirmed its advantages. Recently, a systematic literature review and meta-analysis carried out by Quartley et al. [15] in 2022 compared InterTAN and other available nails, showing that the use of InterTAN nails reduced the risk of revision/reoperation by 64%, implant failure by 62%, and hip and thigh pain by 50% in AO OTA 31-A unstable proximal femoral fractures. Our study confirmed these findings, demonstrating satisfactory clinical and radiological results with long InterTAN nails. We found that all patients achieved union without complications at follow-up, such as varus collapse, implant cut-out, or implant breakage.

A cerclage wire or cable is proposed to improve the accuracy of fracture reduction and construct stability, but its application is still a matter of debate [20]. Numerous anatomical and clinical studies have reported that cerclage wires or cables do not affect the blood supply of the fracture site and that they enhance construct stability [6, 11, 21]. However, some authors remain convinced that these devices increase the surgical time, blood loss, treatment cost, and risk of infection and potential neurovascular damage [7, 10]. Recently, a systematic review and meta-analysis of comparative studies regarding the effect of cerclage wire augmentation on complications, fracture union, and reduction for subtrochanteric femur fractures treated with a femoral nail revealed that there is no statistical advantage in using cerclage wire in relation to the risk of reoperation, nonunion, loss of fixation, and implant failure or the time to union. However, its advantage has been seen in more accurate fracture reduction [20]. We believe cerclage wires are beneficial in some subtrochanteric fracture types, but not all. In our study, cerclage cables were selectively used in 10 patients with associated large butterfly-shaped fragments. We recommended the selective application of a cerclage wire or cable for adjunctive fixation of subtrochanteric fractures in certain fracture types: 1. Large butterfly-shaped fragments 2. Fracture lines involving the lateral wall 3. Fracture displacement after nail insertion or clamp removal.

The limitations of this study include its retrospective design, relatively small sample size, and lack of comparison between different reduction methods and intramedullary nails. Therefore, in the future, we need to increase the sample size and set up a control group to compare the advantages or disadvantages of reduction tools and intramedullary nails.

## Conclusion

Our results indicate that minimally invasive clamp-assisted reduction with long InterTAN nail fixation is encouraging for Seinsheimer Type V subtrochanteric fractures and provides excellent reduction and fixation. Additionally, this reduction technique is simple, reliable, and effective in reducing and maintaining subtrochanteric fractures, particularly when intertrochanteric fractures are irreducible.

## Data Availability

The datasets generated and/or analyzed during the current study are not publicly available due to the data and anonymity of the patients involved but are available from the corresponding author (Yong Wang) at a reasonable request.
